# Reductive metabolites of curcumin and their therapeutic effects

**DOI:** 10.1016/j.heliyon.2020.e05469

**Published:** 2020-11-12

**Authors:** Achyut Pandey, Maya Chaturvedi, Shruti Mishra, Pramod Kumar, Pallavi Somvanshi, Rupesh Chaturvedi

**Affiliations:** a School of Biotechnology, Jawaharlal Nehru University, New Delhi, India; b Department of Biotechnology, TERI School of Advance Studies, New Delhi, 110070, India; c Department of Chemistry, Sri Aurobindo College, University of Delhi, New Delhi, India

**Keywords:** Curcumin, Dihydrocurcumin, Tetrahydrocurcumin, Hexahydrocurcumin, Octahydrocurcumin, Alternative medicine, Biochemistry, Biological sciences, Food chemistry, Food science, Health sciences, Metabolite, Pharmaceutical chemistry, Pharmaceutical science, Pharmacology

## Abstract

Curcumin, a secondary metabolite from the turmeric plant is one of the most promising natural products, which has been studied extensively for decades. It has demonstrated several pharmacological activities *in vitro* and *in vivo*. Various studies have indicated that the pharmacological activity of curcumin is contributed by its metabolites.

The aim of this review is to present an overview of metabolic products of curcumin produced upon its reduction like di, tetra, hexa and octa-hydrocurcumin. In addition, this paper has systematically analyzed the current information regarding medicinal use of reduced metabolites of curcumin and identified the limitations which have hindered its widespread usage in the medical world. Several diverse therapeutic effects have shown to be exhibited by reduced metabolites of curcumin such as antioxidant, anti-cancerous, anti-inflammatory and immunoregulatory activities. The potential underlying molecular mechanisms of the biological activities of reduced metabolites of curcumin have also been highlighted, which may provide insight into the principle of effectiveness of curcumin.

## 1 Introduction

Curcumin is a natural polyphenolic compound which forms a major component of rhizomes from dietary spice turmeric (*Curcuma longa* a plant belongs to *Zingiberaceae* family [1, 2]. It is commonly used as a flavouring agent in various foods and also as a traditional medicinal agent [3, 4].

In several studies, curcumin have shown a diverse range of pharmacological effects like, anti-cancer, anti-oxidation, anti-inflammatory, anti-bacterial activities, free radical scavenging and anti-depression [5, 6, 7]. Owing to these pharmacological effects, curcumin has therapeutic potential over a variety of human diseases such as cancer, cardiovascular disease, diabetes, arthritis, Alzheimer's disease, AIDS, neurological diseases, and Crohn's disease [8, 9, 10, 11]. The Importance of curcumin can be estimated by the fact that thirty-seven cases of clinical trials of curcumin [81] were completed by December 2017 and two cases FDA (Food and Drug administration) clinical phase 4 trials were completed [2, 12, 13].

There are several review articles available which have summarized metabolism of curcumin [12, 13, 14, 15, 16] occuring via oxidation, cleavage, reduction, conjugation methods [12, 87]. Studies have shown that curcumin gets metabolized rapidly in cell culture condition as well as *in vivo*, mainly by reduction and conjugation. Di-, tetra-, hexa- and octahydrocurcumin are formed upon consecutive reduction of the double bonds in the heptadienedione chain of curcumin [17]. Among reduced metabolites, tetra- and hexahydrocurcumin, form the largest portion of curcumin metabolites detected. The diverse biological properties of curcumin are contributed by these reductive metabolites rendering the study of these metabolites critical [15].

This review aims to elucidate the potential relevance of reductive metabolites of curcumin in various diseases and their possible molecular targets (Table 1) and mechanisms underlying curcumin biological effects. Curcumin forms reduced products which are equally important to its pharmacological effects, even in some cases reduced products show more potential than parent compound curcumin, but they are less studied as compare to curcumin [4, 18]. Hence there is need of more comprehensive research in this area to unfold relevant questions like specificity, effectiveness, mechanism of actions, comparative studies of reductive metabolites of curcumin.

**Table-1:** Hydrogenated metabolites and their potential targets.

Curcumin metabolite	Potential targets
DHC	Nrf2, Phospholipase A2, NO, Pi3k/Akt
THC	HDAC1, acetyltrasferase (PCAF), COX-2, caspase-3, SIRT1, SOD2, TGFβ1/Smad3, Protein kinase B/Akt kinase, FOXO, Sir2, ERK, GRASP65, CYP2E1, Keap-Nrf2, IL-4R alpha/JAK1/STAT6, Th2, IL-4, IL-5, gp120-CD4, cyclin D, PCNA, PI3k/Akt/mTOR, HIF-1alpha, p38 MAPK, MDM2, p53, EGFR, pERK1&2, p-AKT, VEGF/VEGFR-2, sterol regulatory element-binding protein 1, peroxisome proliferation-activated receptor gamma, fatty acid synthase, fatty acid binding protein 4.
HHC	COX2, VEGF, Dexamethasone, amyloid precursor protein, β-secretase cleavage enzymes
OHC	P53, MDM2, Bcl-2, Bcl-xl, Bax, Bad, TAK/TAB1, NO, inducible NO synthase, COX-2, Ikβ-α, NF-κβ, Keap-1/Nrf2

## 2 Reductive metabolism

The first time in 1978, Holder *et al.*, published information on the chemical structures of curcumin metabolites, which were isolated from tritium-labeling fate studies in rats. When intraperitoneal and intravenous doses were given to rat, Holder *et al.*, found bile as the main route of elimination of curcumin. The eliminated products consisted of glucuronide conjugates of THC (Tetrahydrocurcumin) and HHC (Hexahydrocurcumin) as primary biliary metabolites and ferulic acid and dihydroferulic acid as minor metabolites [19]. Upon oral administration of curcumin in rats, it was observed that after absorption, less than half of it remained in the lower part of the gut. The retention and absorption in the lower part of the gut may lead to a transformation of curcumin. Instead of curcumin, mainly its glucuronide and sulfate derivatives were detected in urinary excretion, while approximately 40% of the dose was excreted in the unchanged form through faeces over five days. In above mentioned *in vivo* study, curcumin was not detected in heart blood; however, a very less amount was found in the Liver, Kidney, and portal blood [20]. It was only in the early 1980s studies from rat intestine confirmed that curcumin gets transformed during its absorption from the rat intestine [21].

Curcumin is one of the very well-known natural products, known for its various pharmacological activities. However, from the therapeutic perspective, not that much effort has been made in terms of its metabolism. The study of curcumin metabolism may uncover other unexplained advantages of curcumin. Curcumin itself has poor chemical stability, low absorption, and also results in various metabolites and degradation products, which are also less studied areas. Therefore, it can be hypothesized that curcumin's degradation products and its metabolites may be responsible for its immense therapeutic effects. However, this still needs to be adequately validated [13, 22, 23, 24, 25, 26].

Wang *et al.*, used LC-MS/MS method to study pharmacokinetics and tissue distribution of curcumin and its metabolites in mice after intravenous administration of curcumin. Reduced metabolite THC (Tetrahydrocurcumin) and DHC (Dihydrocurcumin) were detected in plasma, and THC was present as a major metabolite. Curcumin was present in the Liver, Kidney, and brain, and DHC and THC were detected in the liver and kidney, respectively. This study concluded that only curcumin could cross the blood-brain barrier and may become a worthy prodrug molecule in the research studies related to neurological disorders such as Alzheimer's disease. Further, the distribution of curcumin and tetrahydrocurcumin in the liver might be playing the role of hepatoprotection, but this needs further studies to get a clearer picture [27].

Later, investigators found curcumin metabolites in subcellular fractions of human and rat intestinal tissue and confirmed that curcumin metabolized in the human intestinal tract. In human intestinal fractions, the extent of curcumin conjugation was much higher than in those from rats; however, in human hepatic fractions, curcumin conjugation was not as much as in those from rats [28].

Curcumin transformed into DHC, THC, and subsequently converted into glucuronide conjugates [29]. In Liver, Kidney, and intestinal mucosa, the conjugative enzyme activity for glucuronidation and curcumin sulfation had also been discovered. Further, a study reported that a significant portion of orally administered curcuminoids were conjugated to glucuronide in the intestine after that, they entered into the portal vein as glucuronide conjugates and further conjugated to form glucuronide/sulfate in the liver. In contrast to intravenously injected curcuminoids, orally administered curcuminoids could not reach the liver in the free form. Concisely, orally administered curcuminoids entered the overall blood circulation in rats and were present primarily as glucuronide and glucuronide/sulfate conjugates [30].

**Figure 1:**
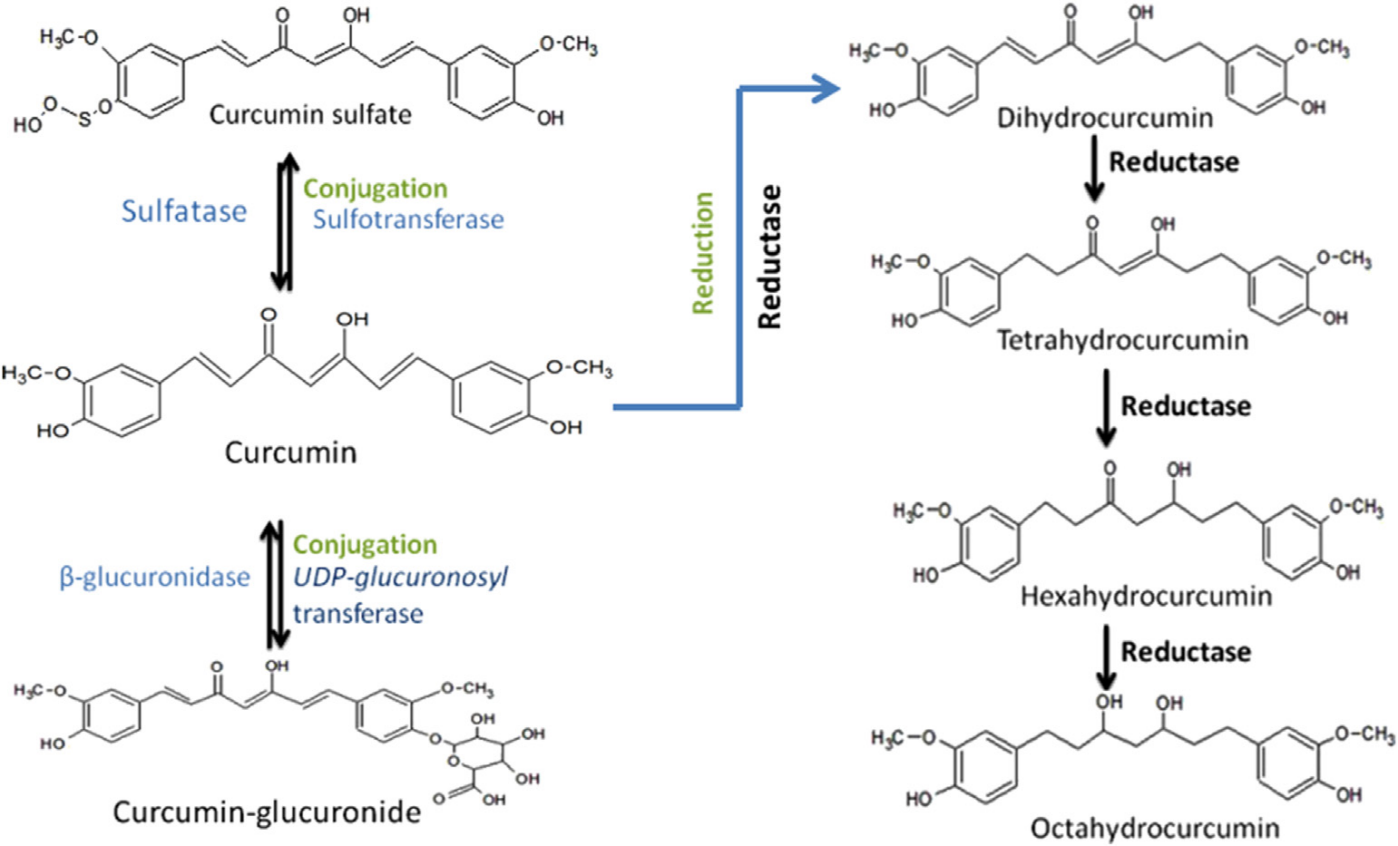
**Conjugation and Reduction of Curcumin:** Curcumin can be conjugated with sulfate moiety by Sulfotransferase and curcumin sulfate can be converted into curcumin by putative enzymes inside the cells. UDP-glucuronosyl transferase can transfer the glucuronol moiety to curcumin and β-glucuronidase can convert curcumin-glucuronide into curcumin. Curcumin can be converted by cellular putative reductase into Di, Tetra, Hexa, and Octahydrocurcumin. These reduced curcumin metabolites can also be conjugated.

Till now, two phases of curcumin metabolism have been reported. Phase-I metabolism involves the reduction of double bonds by which curcumin converted into di, tetra, hexa and octahydrocurcumin. In phase-II metabolism glucuronide or sulfate is conjugated to the curcumin and to its hydrogenated metabolites [28]. Double bond of curcumin is reduced into di, tetra, hexa and finally into octahydrocurcumin by the reductases. Curcumin and its reduced metabolites can be conjugated on any of its phenol-OH site. During conjugation glucuronic acid or sulphate moiety are added by glucuronidases or sulfotransfersaes [31] (Figure 1). An alternative metabolism of curcumin occurs by intestinal microbiota of commensal *Escherichia coli*. CurA, which is a NADPH dependent enzyme in *E. coli* converts curcumin into di and tetrahydrocurcumin [32]. Recently, through one of the studies, the presence of NADPH-dependent reductase CurA enzyme in *vibrio vulnificus* also got highlighted [33].

Studies, as mentioned above, have established physicochemical properties like poor absorption, rapid metabolism, and excretion of Curcumin [34].

## 3 Conjugation

The higher level of conjugation is one of the reasons responsible for the low bioavailability of curcumin. Hydrogenated metabolites of curcumin and curcumin itself undergo the conjugative metabolism process (Figure 1). Glucuronidation and sulfation are the two-phase II metabolism pathways that have been reported so far, in which glucuronidation is primarily followed. Sulfation involves enzymes like human phenol sulfotransferase isoenzymes SULT1A1 and SULT1A3 latter being more efficient [28]. Jamil *et al.*, studied the biotransformation of curcumin in breast cancer cells and the impact of sulfation on cytotoxicity. Curcumin sulfate was found as a main biotransformed product in human breast cancer cell lines. Curcumin sulfate did not stay inside the cell but was excreted quickly into the cellular medium. The sulfonation of curcumin reduces the intracellular concentration of curcumin and thus reduces its growth inhibitory effects [35].

## 4 Functions of reduced metabolites of curcumin

### 4.1 Functions of dihydrocurcumin

The function of the DHC has been less studied. The effect of DHC has been investigated *in vitro* in a human liver cancer cell model for the study of non-alcoholic fatty liver diseases (NAFLD). Hallmarks of NAFLD are oxidative stress, insulin resistance, inflammation andhyperlipidaemia. DHC upregulated Nrf2 to reduce oxidative stress and, may alsoovercome the insulin resistance by increasing the glucose uptake by positively regulating of PI3K/AKT pathway. Moreover, DHC inhibits lipid biosynthesis and increased lipid oxidation in both HepG2 and L02 cells [36]. DHC was also predicted to have a stronger binding affinity than curcumin to the active site of Phospholipase A2 in a molecular docking study suggesting theanti-inflammatory function of DHC [37].

### 4.2 Functions of tetrahydrocurcumin

THC, in comparison to curcumin, was found to be more stable at physiological conditions, more soluble in the aqueous medium, while curcumin more lipid-soluble than THC and more readily absorbed through the gastrointestinal tract in addition to having good stability in the plasma [29, 38, 39], which was the reason why THC was considered as an accessible form of curcumin in vivo [13, 29, 40] Thus, there is a possibility that differential absorption and metabolism of curcumin and tetrahydrocurcumin in cells determine their biological functions. THC demonstrated similar functional activities like curcumin. However, numerous *in vitro* and *in vivo* studies have proved that curcumin possesses better therapeutic properties than THC, including antioxidant, anti-inflammatory, anti-cancer and, anti-viral. A comparative study of activities of both compounds has been summarized by BB Aggarwal *et al.* However, there are some known functional differences between curcumin and THC due to their structural differences. Unlike curcumin, THC lacks α, β dienes that is the reason for its inability to form Michael adducts with intracellular proteins. However, curcumin may disrupt the formation of a disulfide bond by electrophilic dienone. Free thiols on cysteine-rich proteins were available to react with Michael acceptors of curcumin but not with THC [18]. Recently, in contrast to curcumin, THC found incapable of inhibiting HDAC1 (histone deacetylase) and PCAF (acetyltransferase) due to the absence of double bonds, which form Michael acceptor moiety in curcumin. Studies confirmed the previous speculation that THC possesses a different mechanism to deal with cancer than that of curcumin [18].

**Figure 2:**
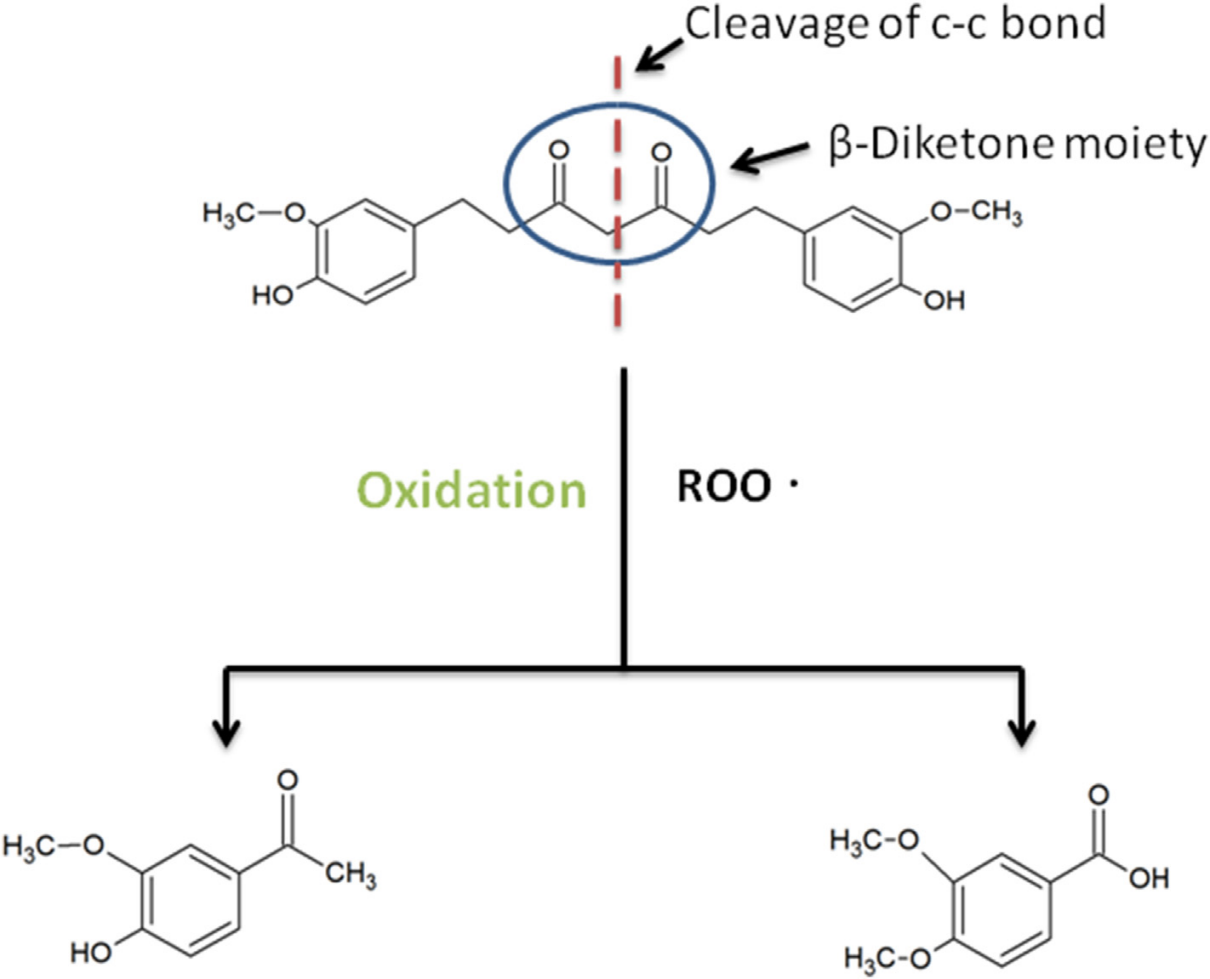
**Free radical scavenging mechanism of tetrahydrocurcumin:** Cleavage at the active methylene group by peroxy radicals (ROO).

Jia-Ching Wu *et al.*, 2014 have summarized therapeutic activities of THC like its antioxidant, anti-neurodegeneration, anti-aging, anticancer effects. Since THC exhibited significant antioxidant activity, it could become a possible medicinal agent to prevent human illness associated with oxidative stress [18]. THC's β-diketone moiety produced an antioxidant response. The oxidation occured by cleavage of the C–C bond at the active methylene carbon between two carbonyls in the β-diketone moiety (Figure 2) [41]. THC demonstrated a neuroprotective effect following traumatic brain injury by acting as an antioxidant and mitochondrial apoptotic pathway inhibitor and by increasing autophagy activation in a rat model [42]. Hyperhomocysteinemia (HHcy) is a risk factor associated with many neurological disorders [43]. THC demonstrated therapeutic effects against Hcy induced mitophagy and mitochondrial dysfunction [44].

THC was shown to suppress the oxidative-stress induced renal damage by acting as a more potent antioxidant agent than curcumin *in vivo* [39]. Tacrolimus is an immunosuppressant drug used in organ transplantation [45]. THC exhibited beneficial effects against tacrolimus induced renal cell damage. Similarly, studies with THC suggested a protective effect against cisplatin-induced nephrotoxicity. Oxidative renal damage was ameliorated through the attenuation of cyclooxygenase-2 and caspase-3 activation in Rat [46]. These were associated with antioxidant and anti-apoptosis effects of THC, indicating that THC could act as an adjuvant therapeutic agent in the treatment of renal damage [47]. The therapeutic potential of THC as a dietary supplement following heavy metal exposure has been summarized by Kukonviriyapan *et al.*, 2016. THC may act as the complimentary chelating agent to enhance the potency of chelators in order to minimize the toxicity of metals. This property of THC may be useful in the treatment of metal induced hypertension and vascular dysfunction [48, 49, 50]. THC improved vascular dysfunction, arterial stiffness, and hypertension associated with cadmium exposure in mice [48]. In another study, THC treatment significantly reversed hepatotoxicity associated with arsenic metal exposure in Rat [51].

THC exhibited anti-fibrotic and anti-diabetic cardiomyopathy effects by suppressing hyperglycemia-induced oxidative stress through the modulation of the SIRT1 pathway. In fact, THC upregulated SIRT1, deacetylated SOD2 expression, and inhibited ROS-induced TGFβ1/Smad3 profibrotic pathway [52]. Further, in rat having chronic kidney disease, THC treatment enhanced the antioxidant protein expression, reduced renal fibrosis, and proteinuria [53]. Besides this, THC also has been implicated as a preoperative measure during pancreatic islets transplantation [54]. The anti-aging activity of THC was shown to be mediated via inhibition of protein kinase B/Akt kinase phosphorylation. Moreover, THC increased the lifespan of Drosophila by suppressing oxidative stress response through the FOXO and Sir2 dependent pathway [55]. THC being an antioxidant, could ameliorate mitochondrial dysfunction associated with ischemia/reperfusion injury by minimizing oxidative stress epigenetically [56]. Furthermore, THC inhibited cerebral ischemia/reperfusion injury-induced ERK and GRASP65 phosphorylation [57].

There are several reports in which THC showed better pharmacological activities than curcumin. THC possessed better hepatoprotective effects and antioxidant activities in case of liver injury than the parent compound curcumin. The activity was mediated via inhibition of CYP2E1 and activation of the Keap1-Nrf2 anti-oxidation pathway [58]. In one of the studies, THC found to be the most potent compound *in vitro* in arachidonic acid-induced blood platelet aggregation when compared with curcumin, bisdemethoxycurcumin, and demethoxycurcumin [59].

Recently, THC has been found to have more potentiality in ameliorating allergic asthma in comparison to curcumin. To be more specific, THC exhibited better inhibitory action against tissue eosinophilia, mucus production, and IL-4Rα/Jak1/STAT6 pathway. Also, only THC and not curcumin attenuated Th cell polarization as Th2 cell subpopulation, Th2 cytokines (IL-4, IL-5), and peripheral eosinophilia. The increased activity of THC attributed to the higher bioavailability of THC in plasma and tissue in comparison to curcumin [51]. One *in vitro* study reported significant wound healing via collagen synthesis by THC [60]. The efficacy of THC nanoparticle as a wound-healing agent has been investigated. THC mPEG-PLGA nanoparticle was able to speed up wound closure *in vitro* and directional migration of human fibroblasts *in vivo* by enhancing collagen deposition and vascular density in wounds. THC lipid nanoparticle found useful in conditions like skin inflammation [61, 62]. After THC encapsulation with hydroxypropyl-cyclodextrins, drug solubility and corneal and retinal epithelial permeability got increased. Increased bioavailability is associated with enhanced antioxidant activity in the ocular epithelial cells, which is responsible for oxidative stress-protective effects in rabbit cornea tissues [63].

THC has the potential to deal with multidrug resistance in cancer in a unique way. It caused cell death through autophagy in Ara-C-resistant acute myeloid leukemia cancer cells [64]. The photoactive metal complex of THC exhibited phototoxicity, which may have potential applications in photodynamic therapy [65]. In a study, THC-loaded vaginal nanomicrobicide demonstrated the gp120-CD4 binding inhibitory ability, which showed its potential in the prophylaxis of HIV-1 infection associated with unprotected sex [66]. Carboxymethylcellulose-THC, a synthetic conjugate prodrug, possessed targeted drug delivery capacity for colon cancer therapy even better than curcumin and THC [67].

Up to 400 mg/kg THC was regarded as safe in rats, as stated in preclinical safety research [68]. THC is widely known as an anticancer agent [69, 70, 71]. As a radiosensitizer, it could stimulate G0/G1 cell cycle arrest by suppressing cyclin D1 and PCNA in glioma cells [69]. THC, in combination with 5-FU, inhibited Oesophageal cell proliferation [70]. THC showed lower inhibitory activity against inflammation-associated carcinogenesis in mice [79]. THC, as a known antitumor compound [72], induced autophagy that was found associated with the suppression of PI3K/Akt/mTOR signaling in A549 lung cancer cells [73]. THC inhibited human breast cancer MCF-7 cell proliferation. The underlying mechanism was that THC accelerated the ROS-dependent mitochondrial apoptosis pathway and arrested the cell cycle at the G0/G1 phase [72]. Antitumor activity of THC is mediated by inhibition of Hypoxia-inducible factor-1α that is associated with increased autophagy and downregulation of Akt/mTOR and p38 MAPK in human osteosarcoma, a malignant bone tumor cell lines. It indicated a relationship between autophagy and tumorigenesis. THC led autophagy induced mesenchymal-epithelial transition and inhibited angiogenesis through HIF-1α. However, the role of other signalling pathways like ERK and JNK needs to be explored [74].

Antitumor activity of THC had been found superior to curcumin in H22 induced ascites tumor mice model. The possible underlying mechanisms that have been suggested were the induction of mitochondria apoptosis pathway, activation of p53, and inhibition of MDM2 [75]. Anticancer activities and mechanisms of THC against cervical cancer have been studied in the tumor mice model. It inhibited tumor growth by inducing cell apoptosis and inhibiting cell proliferation, which was likely to happen through the inhibition of COX-2, EGFR, and p-ERK1&2, and p-AKT [71]. Antitumor angiogenesis activity of THC in Caski-implanted nude mice occurred by inhibiting HIF-1α, microvascular density, VEGF, and VEGFR-2 [76].

THC demonstrated potential pharmacological actions against obesity and non-alcoholic fatty liver diseases [77, 78]. It could suppress oleic acid-induced steatosis in hepatocellular carcinoma cells by modulating several targets like Sterol regulatory element-binding protein 1, Peroxisome proliferator-activated receptor gamma, Fatty acid synthase andFatty acid-binding protein-4 [77].

### 4.3 Functions of hexahydrocurcumin

HHC, a hydrogenated metabolite of curcumin, has been a less studied molecule. It possesses antioxidant, anticancer and cytotoxic, anti-inflammatory, anti-helminths, cardioprotective activities similar to its parent compound curcumin; in some cases, it was found more potent. HHC has shown higher antioxidant activities than curcumin by having more free radical scavenging activities [71]. Further, in the case of lipid peroxidation and red blood cell hemolysis, it also showed more antioxidant activities than curcumin [71]. Studies suggested a relation between structural chemistry and oxidation resistance of HHC, a higher number of hydroxyl groups, phenyl moiety might be responsible for its more potent antioxidant activities [79]. Anticancer activity of HHC has been investigated in the HT29 human colon adenocarcinoma cell line. It inhibited the growth of HT29 cells by downregulating COX-2 expression [80]. Further, the Anti-angiogenesis effect of HHC has been evaluated in Corneal neovascularization disease. The HHC, an inhibitor of COX-2, may play a therapeutic role in corneal neovascularization (CorNV) by acting as an anti-VEGF factor [81]. The role of HHC as a cancer preventive molecule and its effect on cell cycle have been studied in human colon cancer cell SW480. After HHC treatment, the number of viable colon cells decreased, and it also caused an accumulation of cells in the quiescent phase of the cell cycle [82]. Cytotoxic activity and cell cycle analysis of hexahydrocurcumin was studied on SW 480 human colorectal cancer cells [80]. Aberrant crypt foci (ACF) is a characteristic feature in the early phase of colorectal cancer [83]. HHC suppressed the ACF number by becoming a pro-apoptotic agent and inhibited COX-2 expression, at the same time being neutral to COX-1 expression in the Rat [84]. The potential of HHC in Alzheimer's disease to improve memory impairments has been studied in mice. HHC inhibited dexamethasone (a key player in Alzheimer's disease) induced neuronal cell injury by suppressing the expression of amyloid precursor protein and β-secretase cleavage enzyme.

However, the exact mechanism behind this needs to be elucidated to use HHC as a preventive agent in Neurodegenerative diseases.

### 4.4 Functions of octahydrocurcumin

OHC (Octahydrocurcumin) possesses many functions similar to curcumin, in some cases superior to curcumin, like in its properties as antitumor, anti-inflammation [85, 86]. OHC, a final reduced derivative of curcumin, has also been a less studied molecule due to its less accessibility and poor systemic bioavailability. The research suggested that OHC has more superior antitumor activity than its parent compound curcumin [76]. The antitumor effect of OHC and its molecular mechanistic pathway has been investigated in Mouse hepatoma 22 (H22)-induced ascitic tumor-bearing model. OHC targeted major proteins that are involved in cancer cell apoptosis-like p53, murine double minute 2 (MDM2), Bcl-2, Bcl-xl [76]. A study indicated that OHC works via enhancing the mitochondrial apoptotic pathway. OHC enhanced the survival rate of the tumor-mouse model more than that of curcumin. Major morphological characteristics that appear during tumor growth were found to be reduced by OHC. OHC had a more profound effect on H22 cellular apoptosis than curcumin, and it successfully increased the expression of p53, pro-apoptotic molecule Bax, and Bad. Further, OHC was found to inhibit the expression level of MDM2 and anti-apoptotic proteins Bcl-2 and Bcl-xl [85].

The anti-inflammatory role of OHC has been studied in mice models having acute inflammation. The underlying mechanism was that OHC downregulated the NF-kB pathway and inhibited TAK1 phosphorylation and its interaction with TAB1. Inhibitory action of OHC against COX-2 was more pronounced than curcumin [86]. Further, LPS-induced overproduction of NO, inducible NO synthase, COX-2 expression in RAW 264.7 macrophage cells were inhibited by OHC. Degradation of IkB-α was attenuated by OHC that averted the NF-kB translocation to the nucleus [87].

Potential mechanism of action of OHC as a prodrug molecule has been studied in acetaminophen-induced hepatotoxicity and oxidative stress *in vivo*. OHC exhibited better hepato-protection and antioxidant activities than curcumin. The keap1-nrf2 antioxidant pathway was upregulated by suppressing Keap1 expression and blocking the interaction between Keap1-Nrf2. Molecular Docking study indicated that OHC (THC and curcumin also) could bind with Keap1 and to some extent, occupy the Nrf2 binding site and made Nrf2 available to perform its antioxidant activities [58].

### 4.5 Functions of conjugated metabolites

Phenolic glucuronide of curcumin was reported to have some remarkable functional role. It could inhibit microtubule assembly [81]. Curcumin-glucuronide has been reported to involve in mitotic catastrophe and cell death in human cancer cells [88].

Intriguingly, i*n vitro* studies indicated that conjugated curcumin was less effective against tumor cells. However, *in vivo*, these curcumin metabolites contributed significantly to the curcumin's therapeutic activity, as ubiquitously expressed enzymes like β-glucuronidase/sulfatases quickly split the conjugates back into the curcumin parent compound as an activated molecule [89].

Except for curcumin glucuronide, curcumin sulfate, THC, and curcumin have been found to have a significant affinity with the membrane transport proteins named as organic anion-transporting polypeptides which mediate uptake of various natural and synthetic pharmacological compounds [89].

## 5 Conclusions

The findings mentioned in this review have given a summary of how the polyphenol, curcumin derived from *Curcuma longa* plant gets reduced to several metabolites and their respective therapeutic functions. Metabolites of curcumin exhibit diverse biological and pharmacological activities, many of which are distinct from curcumin. In several cases curcumin metabolites have shown more potency than the bioactivities of curcumin, one recently studied example is of octahydrocurcumin, which has higher antitumor activity than the parent compound curcumin.

Reduction of curcumin generates multiple products of curcumin and these products show differential affinity towards number of enzymes and kinases which may be responsible for various pleiotropic effects of curcumin. Reduced metabolites of curcumin have presented therapeutic potential such as antioxidative, anti-cancerous, anti-inflammatory and antiseptic in diseases like liver disorders, neurological, cancer, cardiovascular and lung diseases. More studies are underway to perform the evaluation of preclinical and clinical effectiveness of the curcumin metabolites against human diseases. In comparison to tetrahydrocurcumin, there have been less research on the dihydrocurcumin, hexahydrocurcumin, octahydrocurcumin detail study of which still need to be explored further for their other therapeutic functions. Curcumin forms oxidized and reduced metabolites both, but in this study we have focused about reduced metabolites of curcumin. While there are preclinical studies relating to therapeutic potential of reductive metabolites of curcumin, these need further validations in animal model followed by clinical trials to develop curcumin derived medicinal agents. This review may provide additional insights for researchers, healthcare providers, and other stakeholders to understand pleiotropic effects, molecular targets and their regulatory functions of multicomponent curcumin and their metabolites.

## Declaration

### Author contribution statement

All authors listed have significantly contributed to the development and the writing of this article.

### Funding statement

This research did not receive any specific grant from funding agencies in the public, commercial, or not-for-profit sectors.

### Competing interest statement

The authors declare no conflict of interest.

### Additional information

No additional information is available for this paper.

## References

[1] B.B A., C S., N M., H I. (2007). Curcumin: the Indian solid gold. Adv. Exp. Med. Biol..

[2] Sharma R.A., Euden S.A., Platton S.L., Cooke D.N., Shafayat A., Hewitt H.R. (2004). Phase I clinical trial of oral Curcumin : biomarkers of systemic activity and compliance.

[3] Esatbeyoglu T., Huebbe P., Ernst I.M.A., Chin D., Wagner A.E., Rimbach G. (2012). Curcumin — from molecule to biological function. Angewandte.

[4] Heger M., Golen RF Van, Broekgaarden M., Michel M.C. (2014). The Molecular Basis for the Pharmacokinetics and Pharmacodynamics of Curcumin and its Metabolites in Relation to Cancer S.

[5] Maheshwari R.K., Singh A.K., Gaddipati J., Srimal R.C. (2006). Multiple biological activities of curcumin : a short review. Life Sci..

[6] Aggarwal B.B., Sung B. (2008). Pharmacological basis for the role of curcumin in chronic diseases : an age-old spice with modern targets.

[7] Lim G.P., Chu T., Yang F., Beech W., Frautschy S.A., Cole G.M. (2001). The curry spice curcumin reduces oxidative damage and amyloid pathology in an alzheimer transgenic mouse. J. Neurosci..

[8] Sciences M.L. (2008). Review Curcumin : from ancient medicine to current clinical trials.

[9] Anand P., Kunnumakkara A.B., Newman R.A., Aggarwal B.B. (2007). Reviews bioavailability of Curcumin : problems and promises. Mol. Pharm..

[10] Balasubramanyam K., Varier R.A., Altaf M., Swaminathan V., Siddappa N.B., Ranga U. (2004). Curcumin , a novel p300/CREB-binding protein-specific inhibitor of acetyltransferase , represses the acetylation of histone/nonhistone proteins and histone acetyltransferase-dependent chromatin transcription ∗. J. Biol. Chem..

[11] Venkatesan N., Punithavathi D., Arumugam V. (2000). Curcumin prevents adriamycin nephrotoxicity in rats.

[12] Tsuda T. (2018). Curcumin as a functional food-derived factor: degradation products, metabolites, bioactivity, and future perspectives. Food Funct.

[13] Metzler M., Pfeiffer E., Schulz S.I., Dempe J.S. (2013). Curcumin uptake and metabolism. Biofactors.

[14] Pulido-Moran M., Moreno-Fernandez J., Ramirez-Tortosa C., Ramirez-Tortosa M.C. (2016). Curcumin and health. Molecules.

[15] Prasad S., Tyagi A.K., Aggarwal B.B. (2014). Recent developments in delivery, bioavailability, absorption and metabolism of curcumin: the golden pigment from golden spice. Cancer Res Treat.

[16] Jankun J., Wyganowska-Swiatkowska M., Dettlaff K., JelinSka A., Surdacka A., Watróbska-Swietlikowska D. (2016). Determining whether curcumin degradation/condensation is actually bioactivation. Int. J. Mol. Med..

[17] Zhongfa L., Chiu M., Wang J., Chen W., Yen W., Yee L.D. (2012). Enhancement of curcumin oral absorption and pharmacokinetics of curcuminoids and curcumin metabolites in mice.

[18] Aggarwal B.B., Deb L., Prasad S. (2015). Curcumin differs from tetrahydrocurcumin for molecular targets, signaling pathways and cellular responses. Molecules.

[19] Holder G.M., Plummer J.L., Ryan A.J. (1978). The metabolism and excretion of curcumin (1,7-bis-(4-hydroxy-3-methoxyphenyl)-1,6-heptadiene-3,5-dione) in the rat. Xenobiotica.

[20] Goel A., Kunnumakkara A.B., Aggarwal B.B. (2008). Curcumin as “Curecumin”: from kitchen to clinic. Biochem. Pharmacol..

[21] Anand P., Kunnumakkara A.B., Newman R.A., Aggarwal B.B. (2007). Bioavailability of curcumin: problems and promises. Mol. Pharm..

[22] Karthikeyan A., Senthil N., Min T. (2020). Nanocurcumin: a promising candidate for therapeutic applications. Front. Pharmacol..

[23] Mirzaei H., Shakeri A., Rashidi B., Jalili A., Banikazemi Z., Sahebkar A. (2017). Phytosomal curcumin: a review of pharmacokinetic, experimental and clinical studies. Biomed. Pharmacother..

[24] Zielińska A., Alves H., Marques V., Durazzo A., Lucarini M., Alves T.F. (2020). Properties, extraction methods, and delivery systems for curcumin as a natural source of beneficial health effects. Med.

[25] Kotha R.R., Luthria D.L. (2019). Curcumin: biological, pharmaceutical, nutraceutical, and analytical aspects. Molecules.

[26] Nelson K.M., Dahlin J.L., Bisson J., Graham J., Pauli G.F., Walters M.A. (2017). The essential medicinal chemistry of curcumin. J. Med. Chem..

[27] Wang J., Yu X., Zhang L., Wang L., Peng Z., Chen Y. (2018). The pharmacokinetics and tissue distribution of curcumin and its metabolites in mice. Biomed. Chromatogr..

[28] Ireson C.R., Jones D.J.L., Boocock D.J., Farmer P.B., Gescher A.J., Orr S. (2002). Metabolism of the cancer chemopreventive agent curcumin in human and rat intestine. Cancer Epidemiol. Biomark. Prev..

[29] Pan M.H., Huang T.M., Lin J.K. (1999). Biotransformation of curcumin through reduction and glucuronidation in mice. Drug Metab. Dispos..

[30] Asai A., Miyazawa T. (2000). Occurrence of orally administered curcuminoid as glucuronide and glucuronide/sulfate conjugates in rat plasma. Life Sci..

[31] Dei Cas M., Ghidoni R. (2019). Dietary curcumin: correlation between bioavailability and health potential. Nutrients.

[32] Hassaninasab A., Hashimoto Y., Tomita-Yokotani K., Kobayashi M. (2011). Discovery of the curcumin metabolic pathway involving a unique enzyme in an intestinal microorganism. Proc. Natl. Acad. Sci. U. S. A..

[33] Park S.B., Bae D.W., Clavio N.A.B., Zhao L., Jeong C.S., Choi B.M. (2018). Structural and biochemical characterization of the curcumin-reducing activity of CurA from Vibrio vulnificus. J. Agric. Food Chem..

[34] Wahlstrom B., Blennow G. (1978). Study on fate of curcumin in rat. Acta Pharmacol. Toxicol..

[35] Jamil Q.U.A., Jaerapong N., Zehl M., Jarukamjorn K., Jäger W. (2017). Metabolism of curcumin in human breast cancer cells: impact of sulfation on cytotoxicity. Planta Med..

[36] Yu Q., Liu Y., Wu Y., Chen Y. (2018). Dihydrocurcumin ameliorates the lipid accumulation, oxidative stress and insulin resistance in oleic acid-induced L02 and HepG2 cells. Biomed Pharmacother.

[37] Dileep K.V., Tintu I., Sadasivan C. (2011). Molecular Docking Studies of Curcumin Analogs with Phospholipase A2.

[38] Anand P., Thomas S.G., Kunnumakkara A.B., Sundaram C., Harikumar K.B., Sung B. (2008). Biological activities of curcumin and its analogues ( Congeners ) made by man and Mother. Nature.

[39] Okada K., Wangpoengtrakul C., Tanaka T., Toyokuni S., Uchida K., Osawa T. (2018). Biochemical and Molecular Action of Nutrients Curcumin and Especially Tetrahydrocurcumin Ameliorate Oxidative Stress- Induced Renal Injury in Mice.

[40] Pfeiffer E., Hoehle S.I., Walch S.G., Riess A., Sólyom A.M., Metzler M. (2007). Curcuminoids form reactive glucuronides in vitro. J. Agric. Food Chem..

[41] Sugiyama Y., Kawakishi S., Osawa T. (1996). Involvement of the β-diketone moiety in the antioxidative mechanism of tetrahydrocurcumin. Biochem. Pharmacol..

[42] Gao Y., Zhuang Z., Gao S., Li X., Zhang Z., Ye Z. (2017). Tetrahydrocurcumin reduces oxidative stress-induced apoptosis via the mitochondrial apoptotic pathway by modulating autophagy in rats after traumatic brain injury.

[43] Ansari R., Mahta A., Mallack E., Luo J. (2014). Hyperhomocysteinemia and neurologic Disorders : a review. J. Clin. Neurol..

[44] Endothelial B. (2017). Tetrahydrocurcumin Ameliorates Homocysteine Mediated Mitochondrial.

[45] Fung J.J., Abu-elmagd K., Todo S., Shapiro R., Tzakis A., Armitage J. (2010). NIH Public Access.

[46] Papers O. (2015). Protective Effect of Tetrahydrocurcumin against Cisplatin-Induced Renal Damage: In Vitro and In Vivo Studies.

[47] Park C.S., Jang H.J., Lee J.H., Oh M.Y., Kim H.J. (2018). Tetrahydrocurcumin ameliorates Tacrolimus-induced Nephrotoxicity via inhibiting Apoptosis. Transplantation Proceedings 2018 Nov 1 (Vol. 50, No. 9, pp. 2854-2859).

[48] Sangartit W., Kukongviriyapan U., Donpunha W., Kukongviriyapan V., Surawattanawan P. (2014). Tetrahydrocurcumin Protects against Cadmium-Induced Hypertension , Raised Arterial Stiffness and Vascular Remodeling in Mice.

[49] Kukongviriyapan U., Apaijit K., Kukongviriyapan V. (2016). Oxidative Stress and Cardiovascular Dysfunction Associated with Cadmium Exposure : Beneficial Effects of Curcumin and Tetrahydrocurcumin.

[50] Sangartit W., Pakdeechote P., Kukongviriyapan V., Donpunha W. (2016). Tetrahydrocurcumin in combination with deferiprone attenuates hypertension , vascular dysfunction , barore fl ex dysfunction , and oxidative stress in iron-overloaded mice. Vasc. Pharmacol..

[51] Chen B.L., Chen Y.Q., Ma B.H., Yu S.F., Li L.Y., Zeng Q.X. (2018). Tetrahydrocurcumin, a major metabolite of curcumin, ameliorates allergic airway inflammation by attenuating Th2 response and suppressing the IL-4Rα-Jak1-STAT6 and Jagged1/Jagged2 -Notch1/Notch2 pathways in asthmatic mice. Clin. Exp. Allergy.

[52] Li K., Zhai M., Jiang L., Song F., Zhang B., Li J. (2019). Tetrahydrocurcumin ameliorates diabetic cardiomyopathy by attenuating high glucose-induced oxidative stress and fibrosis via activating the SIRT1 pathway. Oxid Med Cell Longev.

[53] Lau W.L., Singh B., Khazaeli M., Savoj J., Manekia K. (2018). Dietary Tetrahydrocurcumin Reduces Renal Fibrosis and Cardiac Hypertrophy in 5/6 Nephrectomized Rats.

[54] Kim S.S., Jang H.J., Oh M.Y., Lee J.H., Kang K.S. (2018). Apoptosis in Mouse Islets.

[55] Xiang L., Nakamura Y., Lim Y.M., Yamasaki Y., Nose Y.K., Maruyama W. (2011). Tetrahydrocurcumin extends life span and inhibits the oxidative stress response by regulating the FOXO forkhead transcription factor.

[56] Mondal K. (2019). Tetrahydrocurcumin epigenetically mitigates mitochondrial dysfunction in brain vasculature during ischemic stroke. Neurochem. Int..

[57] Lin B.I.N., Yu H., Lin Y., Cai C., Lu H., Zhu X. (2016). Suppression of GRASP65 Phosphorylation by Tetrahydrocurcumin Protects against Cerebral Ischemia/Reperfusion Injury via ERK Signaling.

[58] Luo D.D., Chen J.F., Liu J.J., Xie J.H., Zhang Z.B., Gu J.Y. (2019). Tetrahydrocurcumin and octahydrocurcumin, the primary and final hydrogenated metabolites of curcumin, possess superior hepatic-protective effect against acetaminophen-induced liver injury: role of CYP2E1 and Keap1-Nrf2 pathway. Food Chem. Toxicol..

[59] Chapman K., Scorgie F.E., Ariyarajah A., Stephens E., Enjeti A.K., Lincz L.F. (2019). The effects of tetrahydrocurcumin compared to curcuminoids on human platelet aggregation and blood coagulation in vitro. Thromb. Res..

[60] Trivedi M.K., Gangwar M., Mondal S.C., Jana S. (2017). Protective effects of tetrahydrocurcumin (THC) on fibroblast and melanoma cell lines in vitro: it’s implication for wound healing. J. Food Sci. Technol..

[61] He P., Yan H., Zhao J., Gou M. (2019). An Evaluation of the Wound Healing Potential of Tetrahydrocurcumin-Loaded MPEG-PLA Nanoparticles.

[62] Vandita K., Pal K.I., Pal K.A., Komal S., K S.K. (2018). Accept e us t. Drug Dev. Ind. Pharm..

[63] Maharjan P., Jin M., Kim D., Yang J., Maharjan A. (2019). Evaluation of epithelial transport and oxidative stress protection of nanoengineered curcumin derivative-cyclodextrin formulation for ocular delivery. Arch Pharm. Res. (Seoul).

[64] Tseng Y.H., Lin P.C. (2019). Curcumin and Tetrahydrocurcumin Induce Cell Death in Ara - C - Resistant Acute Myeloid Leukemia.

[65] Alessia Colombo, et al. A highly luminescent tetrahydrocurcumin Ir III complex with remarkable photoactivated anticancer activity. PMID: 30985041 10.1002/chem.201901527 30985041

[66] Mirani A., Kundaikar H., Velhal S., Patel V., Bandivdekar A., Degani M. (2019). Tetrahydrocurcumin-loaded Vaginal Nanomicrobicide for Prophylaxis of HIV/AIDS : in Silico Study , Formulation Development , and in Vitro Evaluation.

[67] Plyduang T., Lomlim L., Yuenyongsawad S., Wiwattanapatapee R. (2014). European Journal of Pharmaceutics and Biopharmaceutics Carboxymethylcellulose – tetrahydrocurcumin conjugates for colon-specific delivery of a novel anti-cancer agent , 4-amino tetrahydrocurcumin. Eur. J. Pharm. Biopharm..

[68] Majeed M., Natarajan S., Pandey A., Bani S., Mundkur L. (2019). Subchronic and reproductive/developmental toxicity studies of tetrahydrocurcumin in rats. Toxicological research.

[69] Zhang X., Peng L., Liu A., Ji J., Zhao L., Zhai G. (2018). The enhanced effect of tetrahydrocurcumin on radiosensitivity of glioma cells. J. Pharm. Pharmacol..

[70] Yang J., Zhong X., Kim S., Kim D., Kim H.S., Lee J. (2018). Comparative effects of curcumin and tetrahydrocurcumin on dextran sulfate sodium-induced colitis and inflammatory signaling in mice. Journal of Cancer Prevention.

[71] Yoysungnoen B., Bhattarakosol P., Changtam C., Patumraj S. (2016). Effects of Tetrahydrocurcumin on Tumor Growth and Cellular Signaling in Cervical Cancer Xenografts in Nude Mice.

[72] Han X., Deng S., Wang N., Liu Y., Yang X. (2017). Inhibitory Effects and Molecular Mechanisms of Tetrahydrocurcumin against Human Breast Cancer MCF-7 Cells.

[73] Song G., Lu H., Chen F., Wang Y., Fan W., Shao W. (2018). Tetrahydrocurcumin-induced autophagy via suppression of PI3K/Akt/mTOR in non-small cell lung carcinoma cells. Mol. Med. Rep..

[74] Zhang Y., Liu Y., Zou J., Yan L., Du W., Zhang Y. (2017). Tetrahydrocurcumin induces mesenchymal-epithelial transition and suppresses angiogenesis by targeting HIF-1α and autophagy in human osteosarcoma. Oncotarget.

[75] Liu W., Zhang Z., Lin G., Luo D., Chen H., Yang H., Liang J., Liu Y., Xie J., Su Z., Cao H. (2017). Tetrahydrocurcumin is more effective than curcumin in inducing the apoptosis of H22 cells via regulation of a mitochondrial apoptosis pathway in ascites tumor-bearing mice. Food & function.

[76] Yoysungnoen B., Bhattarakosol P., Patumraj S., Changtam C. (2015). Effects of Tetrahydrocurcumin on Hypoxia-Inducible Factor-1 ? and Vascular Endothelial Growth Factor Expression in Cervical Cancer Cell-Induced Angiogenesis in Nude Mice.

[77] Chen J., Kong Z., Tsai M., Lo C., Ho C., Lai C. (2018). ScienceDirect Tetrahydrocurcumin ameliorates free fatty acid-induced hepatic steatosis and improves insulin resistance in HepG2 cells. J. Food Drug Anal..

[78] Pan M., Chen J., Kong Z., Wu J., Ho C., Lai C. (2018). Attenuation by tetrahydrocurcumin of adiposity and hepatic steatosis in mice with high-fat-diet-induced obesity. J. Agric. Food Chem..

[79] Huang Y., Cao S., Zhang Q., Zhang H., Fan Y., Qiu F. (2018). Biological and pharmacological effects of hexahydrocurcumin, a metabolite of curcumin. Arch Biochem Biophys [Internet].

[80] Srimuangwong K., Tocharus C., Chintana P.Y., Suksamrarn A. (2012). Hexahydrocurcumin enhances inhibitory effect of 5-fluorouracil on HT-29 human colon cancer cells.

[81] Chen C.K.C., Huang S.C.J., Lai L.L.C., Lee C.H.C., Chen C. (2017). Anti-angiogenic effect of hexahydrocurcumin in rat corneal neovascularization. Int. Ophthalmol..

[82] Chen C.Y., Yang W.L., Kuo S.Y. (2011). Cytotoxic activity and cell cycle analysis of hexahydrocurcumin on SW 480 human colorectal cancer cells. Nat Prod Commun.

[83] Langner C. (2015). Serrated and Non-serrated Precursor Lesions of Colorectal Cancer.

[84] Srimuangwong K., Tocharus C., Tocharus J., Suksamrarn A. (2012). Effects of hexahydrocurcumin in combination with 5-fluorouracil on dimethylhydrazine-induced colon cancer in rats.

[85] Zhang Z., Luo D., Xie J., Lin G., Zhou J., Liu W., Li H., Yi T., Su Z., Chen J. (2018). Octahydrocurcumin, a final hydrogenated metabolite of curcumin, possesses superior anti-tumor activity through induction of cellular apoptosis. Food & function..

[86] Zhang Z.B., Luo D.D., Xie J.H., Xian Y.F., Lai Z.Q., Liu Y.H. (2018). Curcumin’s metabolites, tetrahydrocurcumin and octahydrocurcumin, possess superior anti-inflammatory effects in vivo through suppression of TAK1-NF-κb pathway. Front. Pharmacol..

[87] Zhao F., Gong Y., Hu Y., Lu M., Wang J., Dong J. (2015). Curcumin and its major metabolites inhibit the inflammatory response induced by lipopolysaccharide: translocation of nuclear factor-κB as potential target. Mol. Med. Rep..

[88] Dempe J.S., Pfeiffer E., Grimm A.S., Metzler M. (2008). Metabolism of curcumin and induction of mitotic catastrophe in human cancer cells. Mol. Nutr. Food Res..

[89] Jaerapong N., Jamil Q.A., Riha J., Milovanovic D., Krupitza G., Stieger B. (2019). Organic anion-transporting polypeptides contribute to the uptake of curcumin and its main metabolites by human breast cancer cells: impact on antitumor activity. Oncol. Rep..

